# Nanocomposites for Improved Physical Durability of Porous PVDF Membranes

**DOI:** 10.3390/membranes4010055

**Published:** 2014-02-24

**Authors:** Chi Yan Lai, Andrew Groth, Stephen Gray, Mikel Duke

**Affiliations:** Institute for Sustainability and Innovation, College of Engineering and Science, Victoria University, P.O. Box 14428, Melbourne, Victoria 8001, Australia; E-Mails: chi.lai2@live.vu.edu.au (C.Y.L.); andrew.groth@evoqua.com (A.G.); stephen.gray@vu.edu.au (S.G.)

**Keywords:** poly(vinylidene fluoride), nanoclay, nanocomposite membranes, microfiltration, ultrafiltration, abrasion resistance, physical durability

## Abstract

Current commercial polymer membranes have shown high performance and durability in water treatment, converting poor quality waters to higher quality suitable for drinking, agriculture and recycling. However, to extend the treatment into more challenging water sources containing abrasive particles, micro and ultrafiltration membranes with enhanced physical durability are highly desirable. This review summarises the current limits of the existing polymeric membranes to treat harsh water sources, followed by the development of nanocomposite poly(vinylidene fluoride) (PVDF) membranes for improved physical durability. Various types of nanofillers including nanoparticles, carbon nanotubes (CNT) and nanoclays were evaluated for their effect on flux, fouling resistance, mechanical strength and abrasion resistance on PVDF membranes. The mechanisms of abrasive wear and how the more durable materials provide resistance was also explored.

## 1. Introduction

### 1.1. The Water Issue and Role of Membrane Technology

Water is becoming scarcer due to droughts brought on by climate change, as well as increased pressures on water sources due to increased urbanization and population growth. Water is essential for human life and culture, and is thus a critical resource for our sustainable future. To address the shortages and ensure water security, there is a rapid rise in the demand for new sources (e.g., sea and groundwater desalination) and the use of recycled water to replace more valuable potable water. A key technology in our ability to access new water sources is membranes [1].

The development of membrane technology has occurred over 150 years [2,3]. It started as a research interest with limited large scale applications. A key milestone for this technology was the development of a reverse osmosis (RO) membrane in the late 1950s [4,5] using cellulose acetate, which showed high salt rejection and high fluxes and thus was promising for membrane seawater desalination process. RO technologies have been commercialized since 1964 [6]. With continual development of other membrane materials, large scale application of membrane filtration to produce high-quality drinking water commenced in the mid-1980s. The first large scale use of microfiltration/ultrafiltration plant was established in Saratoga, CA, USA in 1993 with a capacity of 3.6 million gallons per day [7].

The most common applications include producing valuable water from seawater and brackish water (desalination), as well as treating industrial wastewaters (desalination and filtration). Membrane systems are used to replace processes, such as secondary sedimentation, flocculation, settling tanks, and granular filtration [8], that are usually found in conventional water treatment plants. One of the major advantages of incorporating a membrane system is that a reduced amount of chemicals that are used in the treatment process. Membrane systems also have smaller footprint and consistently produce high quality water. The ability to rapidly and continuously remove salt, contaminants and pathogens makes membrane technology attractive [9].

**Figure 1:**
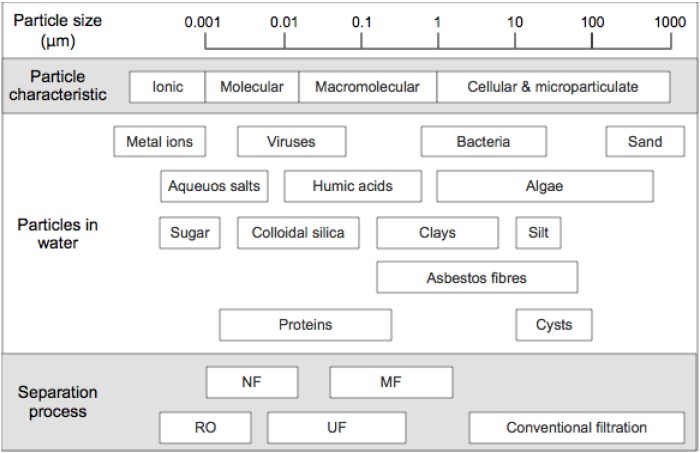
Typical membrane processes and applications (adapted from [9]).

The general classification of membrane types in order of decreasing pore size is microfiltration (MF), ultrafiltration (UF), nanofiltration (NF) and RO. MF and UF membranes are used as an advanced water treatment process for removing particles including silt and pathogens [9], while RO and NF are typical processes for desalination of saline water (e.g., seawater). Figure 1 summarizes the application range of the typical membrane processes. The operating pressure for each membrane application is generally based on the pore size of the membranes; typically low pressure range of for MF and UF (1–2 bar and 2–10 bar) and higher pressure required for NF and RO (7–14 bar and 10–70 bar) [9].

Membranes can be made of polymers or inorganic materials (ceramic, carbon or metal) [10]. The most commonly considered for water treatment are polymeric and ceramic. Commonly used materials for ceramic membranes include aluminium oxide (Al_2_O_3_), titanium dioxide (TiO_2_), zirconium dioxide (ZrO_2_), silicon dioxide (SiO_2_), or their combinations, to achieve the desired filtration mode and performance [11] and can carry out MF and UF. Ceramic membranes show better performance than polymer membranes in applications that require superior physical, chemical and thermal stability [12]. However, ceramic membranes are generally more expensive, brittle and difficult to produce [13]. Polymeric membranes, are lower cost, and flexible, and are widely adopted in the water industry. They usually come in the format of flat sheet or hollow fibre, where hollow fibre is the most popular membrane format for water treatment applications.

Despite the success of polymer membranes in treating waters today, the conventional polymeric materials is limiting them from their ability to treat waters that are major contributors to membrane fouling or require a higher physical durability [1]. Addressing these issues are essential for the growth of membrane applications and its future development. One avenue that can be considered is a composite membrane material, which combines the physical durability of ceramics with the low cost virtues of polymers.

### 1.2. Current Performance Issues

#### 1.2.1. Membrane Fouling

Membrane fouling is one of the major problems encountered in the water industry. Fouling hinders the flux of clean water through the membrane [14] which leads to an increase in feed pressure and requires frequent cleaning of membranes. Other than that, membrane life is also reduced due to biological growth, physical pore blocking and polymer degradation. With established membrane processes today, a portion of the fouling can be reversed by flow management including backwashing and flow relaxation. Additional cleaning protocols such as air scouring and chemical cleaning are also implemented to control fouling. Although these are quite effective in practice, there is an irreversible component which eventually requires membranes to be replaced.

Fouling may be caused by one or more of the following: particulate deposition, adsorption of organic molecules, inorganic deposits as well as microbial adhesion and growth [9]. The foulants, mechanism and the mitigation of each type of fouling are summarised in Table 1.

**Table 1:** Properties of various fouling types [9,15,16,17,18,19,20].

Fouling type	Foulants	Mechanism	Mitigation
Particulate deposition 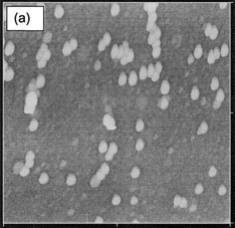 (Reprinted with permission from [17]. Copyright 2001 Elsevier)	Inorganic particles and colloids from weathering of rocks (e.g., silts and clays)	Deposition of particles and colloids forms cake layer on top of membrane which become compressed and reduce flux	Backwashing or air scrubbing is often effective to remove the cake
Organic fouling 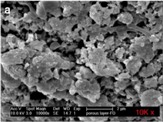 (Reprinted with permission from [18]. Copyright 2013 Elsevier)	Natural organic matters (NOM) including humic acids, fulvic acids, proteins, amino sugars, polysaccharides, polyoxyaromatics	Negative charged foulants have an affinity for charged membrane surface which forms layer reducing flux and salt rejection	Chemical cleaning with caustic and/or chlorine is used to control organic fouling
Inorganic fouling 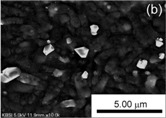 (Reprinted with permission from [19]. Copyright 2013 Elsevier)	Inorganic precipitates such as metal hydroxides	Accumulation of inorganic precipitates causes scaling on membrane surface or within pore structure	Cleaning with acids and chelating agents can remove scales and metal dioxides from fouling layers
Biofouling 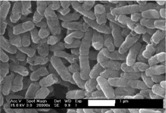 (Reprinted with permission from [20]. Copyright 2007 Elsevier)	Microorganism including bacteria, algae and fungi	Microbial activities lead to formation of biofilms on the membrane	Biofouling is commonly controlled using chlorine (including chloramine) and biocide cleans

Of the four types of fouling evaluated, organic fouling caused by NOM is the most challenging type to mitigate due to the complexity and varied composition of NOM. Other than using real wastewater samples, model foulant solutions made up by bovine serum albumin (BSA) and sodium alginate, which represent the predominant organic foulant types proteins and polysaccharides respectively, have been widely adopted in fouling testing [21].

Membrane fouling remains a major challenge to overcome for the water industry due to its complex mechanism and varieties. Besides conventional mitigation methods like backwashing and chemical cleaning, there has been some interest in improving low pressure membrane anti-fouling performance by incorporating nanoparticles into the membranes [22,23,24,25,26,27,28]. While progress into anti-fouling properties has been made via nanoparticles, it is also important to explore current research aiming to improve physical durability.

#### 1.2.2. Physical Durability

Conventionally, pretreatment of seawater is achieved via coagulation/flocculation followed by granular media or dual media filtration. Such pre-treatment is necessary to remove particles, silt, colloids and micro-organisms before the water is treated by RO so as to reduce fouling on the RO membranes. Recently pretreatment with low pressure MF and UF membranes has become more popular and seawater desalination plants that have adopted UF pretreatment include those in Adelaide (Australia), Perth (Australia), Yu-Huan (China), Fukuoka (Japan), Saudi Arabia and Turkey [29]. Filtration by MF or UF removes a wider spectrum of particles [30] than conventional coagulation/filtration and the improved water quality subsequently reduces RO fouling and cleaning frequency. Other than having smaller plant footprint size which reduces capital investment [31], MF/UF pretreatment uses fewer chemicals compared to coagulation and flocculation ahead of dual media filtration [32].

**Figure 2:**
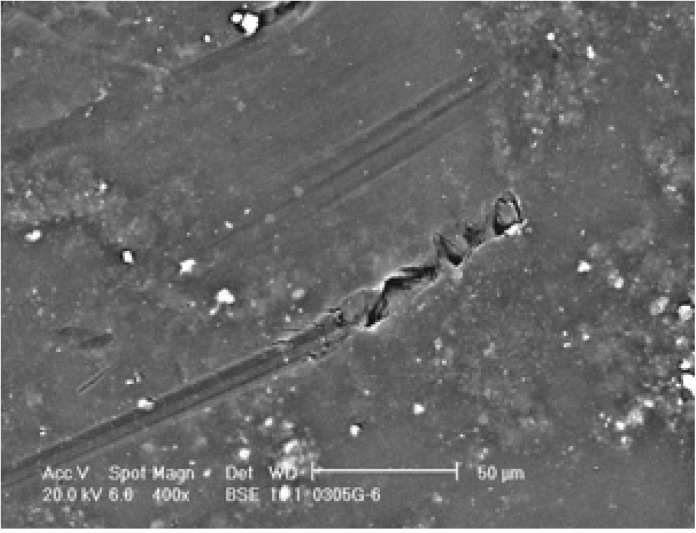
Sand particle abrasion (Reprinted with permission from [38]. Copyright 2011 Genesys).

While these benefits and the technical and economic feasibility of MF/UF pretreatment have been demonstrated in field studies [31,33,34], conventional granular media filtration still remains the pretreatment process for medium and large size desalination plants. One reason for this is the shortened lifespan of MF/UF membranes treating seawater compared to wastewaters and surface waters. Lifetimes of only 3–5 years are achieved for seawater applications [35] compared to 7–10 years for water and wastewater applications [36]. This shorter life expectancy is likely to be related to the harsher condition provided by the water source. Surface water and wastewater contain bio/organic particles, while harder and more abrasive particles including sand and silica based debris are present in seawater [37]. This discrepancy in contaminant characteristics between water sources is likely to be the reason for the shorter life expectancy of polymer MF/UF membranes in seawater, and is supported by an autopsy of RO membranes that identified abrasion with biofouling as the leading cause (28%) [38]. Figure 2 shows membrane surface damage caused by sand particle abrasion. Further, the quality of the seawater fed to a desalination plant is subject to the location of the intake. In general, MF and UF pretreatment are associated with cleaner seawaters taken from costly deep offshore intakes, and avoiding the poorer water qualities associated with shallow, near shore intakes [39,40]. MF/UF filtration membranes with stronger abrasion resistance may relax the costly need for deeper offshore intakes and/or offer more options to the types of water suitable for desalination plants with MF/UF pretreatment.

Microscreening with mesh size of 120 μm or less is currently installed ahead of the membranes [39] to prevent damage from shells and other abrasive particles in seawater. This increases both the capital and running costs, and abrasive particles smaller than the screen mesh size, such as clay/silt aggregates are in the range of 1–40 μm and that of phytoplankton ranges from 4 to 120 μm [41], still can accelerate the wear of membranes. Additionally, some algae and diatoms with exoskeletons made of silicon or calcite are less than 5 μm in size [38] and have low removal efficiencies by screening. Therefore increased abrasion from seawaters appears unavoidable and improved physical endurance of the MF/UF membranes is required to improve their in service life.

Other than abrasive wear, fibre breakage is another durability issue for MF/UF membranes which leads to loss of membrane integrity. Fibre breakage can be attributed to membrane stress and strain from operating conditions, including backwashing or excessive movement owing to vigorous bubbling [42]. Study on commercial membranes based on data obtained from the literature, membrane manufacturers and water treatment plants showed that the annual fibre failure rate was between 1 and 10 per million fibres [43]. Although this failure rate was acceptable based on the overall satisfactory microbiological filtration performance, further reducing this rate can lower replacement costs and down-time. To reduce fibre breakage, one of the keys is to improve the mechanical properties including tensile strength and stiffness of the membrane materials [43,44].

There has been little published work focused on improving MF/UF physical durability, but nanocomposite inorganic/polymer materials are known to have improved physical performance over polymers [45] and fabrication of nanocomposite MF/UF membranes may be a means to achieve greater service lifetimes.

### 1.3. Polymer Composite and Nanocomposite

In order to gain the specialised benefits of both organic and inorganic materials, inorganic materials can be included into the polymer matrix. Fillers in the micrometre scale, including calcium carbonate, glass beads and talc, are commonly used in conventional polymer composites that are commercially available. By modifying their volume fraction, shape and size, various mechanical properties of the composite materials are enhanced [46]. Composites incorporating short glass fibres which have a high aspect ratio (ratio of length to thickness) have been reported to improve mechanical performance such as fatigue strength and tensile strength [47,48]. More recently, fillers including layered silicates and carbon nanotubes (CNT) have demonstrated similar mechanical improvement with considerably lower loading given their large aspect ratio at the nanometre scale [46].

These nanofillers can be incorporated into membranes producing a new class of membranes known as nanocomposite membranes. They are receiving increasing attention worldwide including from the water treatment industry. Polymer membranes incorporated with TiO_2_ were often reported with increased hydrophilicity and improved antifouling behaviour [23,49]. Increased in flux was reported for membranes containing metal-organic framework (MOF) nanoparticles [50,51]. As for silver nanoparticles, Lee *et al*. [52] utilised them as a biocide to inhibit microbial fouling on polyamide membranes, with results demonstrating a substantial anti-fouling property borne from the inclusion of the silver nanoparticles. The mechanical enhancement observed in conventional composite materials also has great potential in increasing the robustness of MF and UF membranes.

Given the wide varieties of nanofillers available and their abilities to enhance different properties, careful selection of suitable nanofillers and exploring how nanocomposites are developed for membranes could possibly improve the durability of low pressure filtration membranes.

## 2. Nanocomposite Membranes

### 2.1. Membrane Materials

At the same time as improving durability of polymer materials to be applied as water treatment membranes, the material must also possess the essential pore size feature that gives its ability to operate as a membrane. Typical polymers used for low pressure membranes in the water industry include polypropylene (PP), polytetrafluoroethylene (PTFE), polysulfone (PSf) and poly(vinylidene fluoride) (PVDF) [53,54].

PVDF has been chosen as the focus of this review as it is the most popular UF material among membrane manufactures. PVDF plays an important role in various industries, such as pulp and paper, nuclear-waste processing and chemical processing [55], owing to its remarkable chemical and physical properties. Its strong chemical resistance against corrosive chemicals including acids, bases, oxidants and halogens [56] makes it an excellent polymeric membrane material and popular among various research groups. As membranes, it is the most widely used in water treatment for the same reasons but in a hydrophilic form, and also has the ability to be controllably porous for MF and UF application. PVDF MF and UF membranes are usually prepared by phase inversion [9,57] which is the most common technique for commercial fabrication of MF/UF membranes. PVDF is a crystalline polymer which can add a degree of complexity to the fabrication process and its various crystalline phases often associate with changes in material properties that must be explored. Efforts to improve physical durability on PVDF membranes using various nanofillers are likely to reach in to many more water treatment applications.

#### PVDF Crystalline Phases

**Figure 3:**
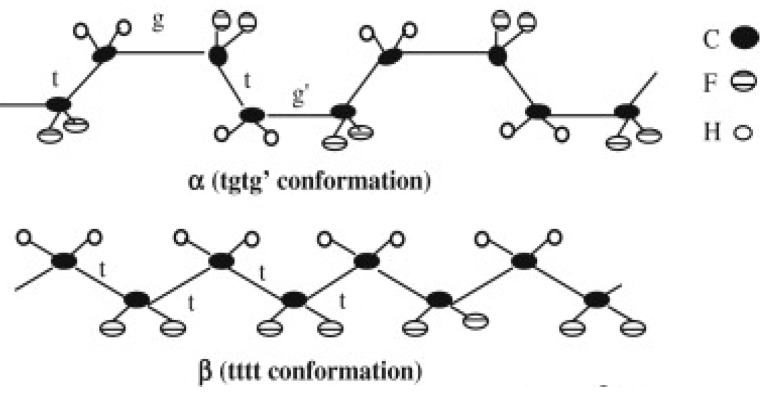
Conformation of PVDF α- and β-phase (Reprinted with permission from [64]. Copyright 2009 Elsevier).

Among the five phases of PVDF, namely α, β, γ, δ and ε [56], α- and β-phase are the most reported in the literature [58]. While α-phase is kinetically favourable owing to a trans-gauche configuration, β-phase has all-trans conformation which is the most thermodynamically stable form (Figure 3) [59]. β-phase also exhibits the most activity for piezo/pyroelectric properties [60,61] which is good for electromechanical and electroacoustic transducer applications. Furthermore, previous studies [55,62] have identified that shifting from α-phase to β-phase is related to an improvement in abrasion resistance and mechanical properties such as stiffness and toughness in nanocomposite materials. As the β-phase has these attractive properties, studies were carried out to investigate ways of shifting PVDF crystalline phase from α to β. Particularly for membranes, these methods include: incorporating nanoparticles such as CNT [57]; decreasing the temperature of the coagulation bath [63] and changing the coagulation bath medium from water to C1–C8 alcohols [58].

### 2.2. Nanofillers

#### 2.2.1. Nanoparticles

**Figure 4:**
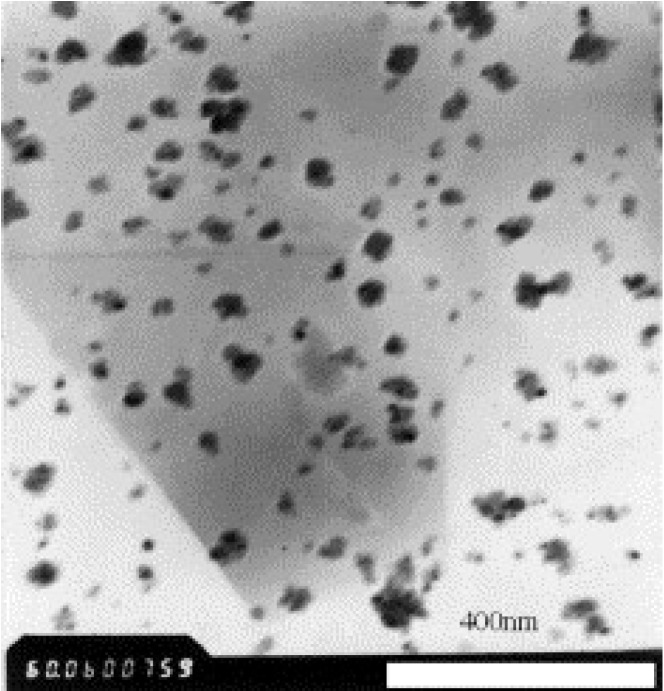
TEM image of TiO_2_ nanoparticles (Reprinted with permission from [65]. Copyright 2005 Elsevier).

Inorganic nanoparticles such as Al_2_O_3_, TiO_2_ (Figure 4), ZrO_2_, SiO_2_ and zinc oxide (ZnO) can be used for reinforcing or toughening polymeric materials [46]. Recently, these particles were incorporated into PVDF membranes and the effect on membrane properties including mechanical enhancement, hydraulic performance and fouling resistance was evaluated.

##### 2.2.1.1. Mechanical Enhancement

PVDF membrane incorporated with 40 wt % of TiO_2_ was tested for mechanical resistance for the use in vapour permeation processes. It demonstrated stronger resistance to compaction than pure PVDF membrane under pressure of 30 bar as shown from the decrease of pore volume percentage of 17% compared to 83% [66].

**Figure 5:**
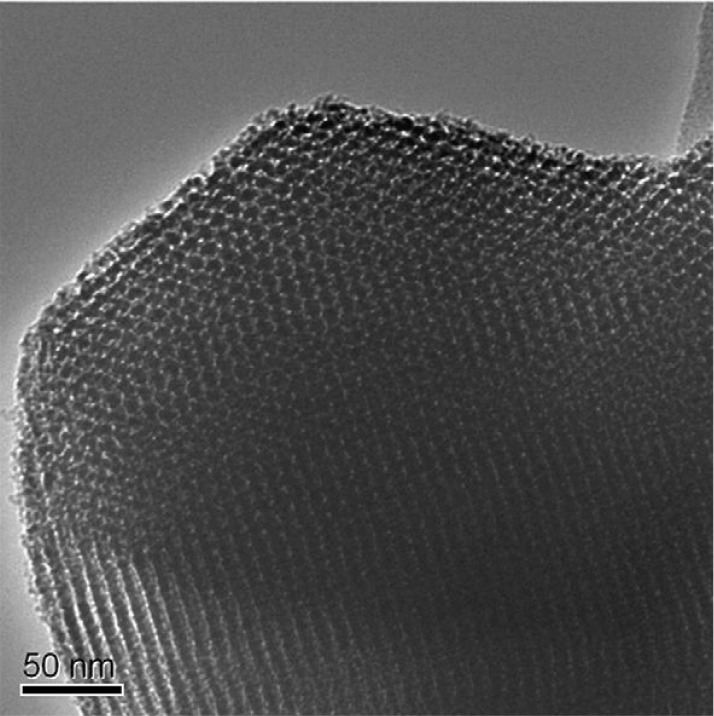
TEM image of SBA-15 particles (Reprinted with permission from [22]. Copyright 2010 Elsevier).

Nanoparticles also have potential for improving membrane mechanical properties in UF applications shown by tensile testing. In particular, PVDF flat sheet membrane with 0.54 wt % of SBA-15 (a mesoporous silica material with a highly ordered 2D hexagonal mesostructure and thick uniform silica walls as shown in Figure 5) increased tensile strength from 0.151 MPa to 0.183 MPa while that loaded with 0.36 wt % increased elongation-at-break from 22.6% to 49.4% [22]. This showed that both stiffness and ductility of the nanocomposite membrane were improved. Likewise, PVDF nanocomposite membranes incorporated with ZnO nanoparticles exhibited increased tensile strength and elongation-at-break [28]. The strength was enhanced especially at high ZnO loading where the tensile strength was twice that of the unmodified membrane when the ZnO to PVDF ratio was at 3:15 and 4:15. PVDF/TiO_2_ hollow fibre membranes prepared by either TiO_2_ sol-gel or blending method showed 30% increase in tensile strength [26]. However, elongation at break decreased from 162% to 120% likely due to the rigidity of the inorganic particles. Han *et al*. [67] explored the effect of using multiple types of nanoparticles (TiO_2_, SiO_2_ and Al_2_O_3_) in PVDF hollow fibre membranes. It was noted that all nanocomposite membranes had higher tensile strength, and the best improvement was from 1.71 MPa to 3.74 MPa with a combination of 2 wt % TiO_2_ and 1 wt % Al_2_O_3_. The improvement could be attributed to the reduced macrovoid formation observed in the nanocomposite membranes. Yet, ductility of the composite membranes was reduced compared to the neat PVDF membrane. The authors suggested the decrease was due to the brittleness of the particles compared to the more flexible polymer chain. However, the decreased ductility could be owing to the increased cross-linking arising from the nanoparticle inclusion rather than the brittleness of the particles, as the loads used for the tensile testing are of a magnitude likely to break the polymer-nanoparticle bonds but not the particles themselves.

##### 2.2.1.2. Hydraulic Performance

Increase in water flux has been observed with PVDF membranes incorporated with TiO_2_ [26,67,68], SiO_2_ [25,69] and SBA-15 [22]. This was associated with changes in hydrophilicity, surface pore size and porosity. Hydrophilicity of the PVDF material was reported to be associated with its crystalline phase and polarity [70]. PVDF/SiO_2_ hollow fibre membranes prepared by Yu *et al*. [25] showed the PVDF β-phase crystallinity was directly proportional to the membrane hydrophilicity as measured by X-ray power diffraction (XRD) and contact angle respectively as shown in Table 2. The greater hydrophilicity was one of the reasons for increased in water permeability. Composite membranes also demonstrated better rejection rates in waste water treatment [22,68]. Here, the PVDF composite membrane with 1.95% of TiO_2_ (by weight of dope) tested with oil refinery wastewater showed 99% rejection compared to 63% by neat PVDF membrane [68]. It was likely because of the more hydrophilic membrane surface which repelled the oily components in the wastewater.

**Table 2:** Properties of PVDF/SiO_2_ hollow fibre membranes (arranged according to the intensity of the β-phase peak; largest on top). Reproduced with permission from [25]. Copyright 2009 Elsevier.

Membrane No.	SiO_2_ concentration (wt % in dope)	Contact angle (°)	Pure water flux (L/m^2^·h)
MTEOS-3	3	53.4	301
MTEOS-2	2	64.4	255
MTEOS-4	4	67.7	210
MTEOS-1	1	78.5	185
MTEOS-5	5	76.3	125
MTEOS-0	0	82.9	80

##### 2.2.1.3. Fouling Resistance

Neat PVDF and PVDF/TiO_2_ (2 wt % in dope) membranes were tested with 100 ppm of casein at constant pressure [23]. The composite membrane had a lower modified fouling index (MFI) which showed that TiO_2_ improved membrane fouling resistance. Indicated by a decrease in the ratio of permeate flux decline (flux ratio from start of filtration with foulant solution to stable permeate flux), various kinds of nanoparticles including SBA-15 [22], SiO_2_ [25] and TiO_2_ [26] had the potential to improve the anti-fouling properties of PVDF membranes. Again, the improved fouling resistance was likely related to the higher hydrophilicity influenced by the crystalline phase [25,26]. To examine the membrane resistance to irreversible fouling, fouling experiment using synthetic wastewater containing sodium alginate, humic acid and BSA with physical cleaning between each filtration cycle was conducted [28]. All modified PVDF/ZnO membranes were able to restore the initial flux after physical cleaning for the four filtration cycles while the neat PVDF membrane showed continuous decline of the initial flux. This inferred that the foulants were less likely to attach to the surface of the nanocomposite membranes, thus improving their resistance to irreversible fouling. While these composite membranes showed enhanced antifouling properties, only a few studies [22,25,28] included additives such as poly(vinyl pyrrolidone) (PVP) and poly(ethylene glycol) (PEG) in the membrane formulation. These additives are common in commercial PVDF membranes and act as pore forming agent and control the membrane surface properties [71]. As such, fouling studies on nanocomposite materials should be evaluated with the presence of these polymer additives.

#### 2.2.2. CNT

CNT are allotropes of carbon with cylindrical shape and they can be classified into single-walled nanotubes (SWCNT), multi-walled nanotubes (MWCNT) and carbon nanofibers (CNF) [46]. CNT often have excellent strain to failure and stiffness, making them good reinforcement for polymers. PVDF/MWNTs nanocomposites prepared with solvent evaporation have shown higher tensile strength and Young’s modulus [72,73].

In the membrane context, Mago *et al*. [57] prepared PVDF nanocomposites membranes by phase inversion with ethanol or water as the non-solvents. 5 wt % MWCNT were used in this study. The addition of MWCNT and the use of ethanol as the non-solvent increased β-phase crystallization of the PVDF. In contrast, without incorporating MWCNT or using water as the non-solvent resulted in crystallization of the PVDF mainly in the α-phase.

Compared to other types of nanofillers, the effect of CNT on PVDF membrane properties has not been widely explored and the high cost and limited commercial availability of CNT also reduces their potential for low cost nanocomposite membranes.

#### 2.2.3. Nanoclay

**Figure 6:**
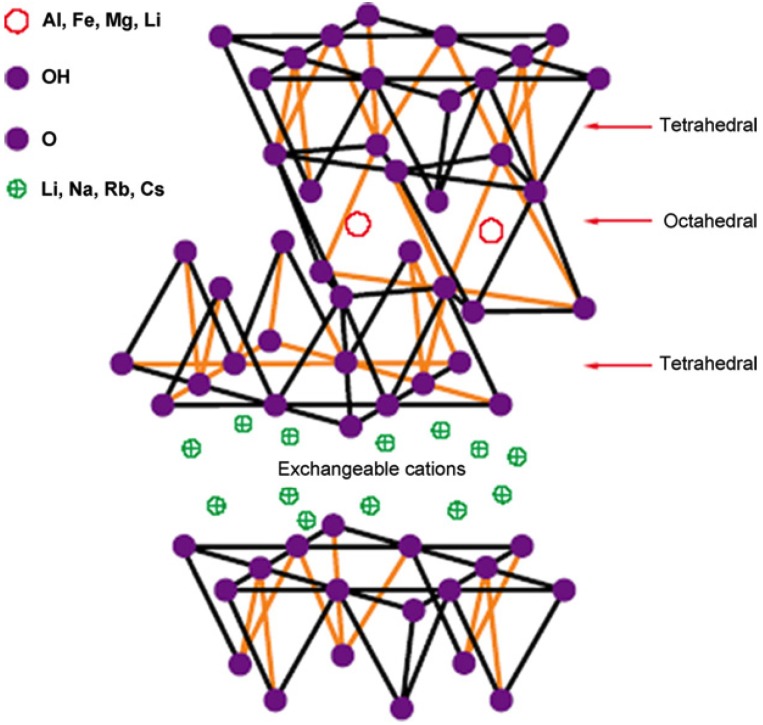
The structure of 2:1 layered silicate (Reprinted with permission from [80]. Copyright 2002 Elsevier).

Nanoclay, which is of relatively low cost and commercially available [46], has been widely investigated as a nanofiller for nanocomposite materials which have enhanced mechanical properties [55,74,75,76,77] and abrasion resistance [62,78,79] in uses including engineering applications, car manufacturing and food packaging industries. These improvements are associated with nanoclay acting as a reinforcing agent as well as changing the PVDF crystalline phase [55,62]. Nanoclay has a layered silicate structure as show in Figure 6, where its thickness is about 1 nm while its width and length can be up to hundreds of nm [80]. Without modification, nanoclays, such as montmorillonite (MMT), have hydrophilic properties. In order to increase its compatibility with the polymeric material, it is often surface modified with organic surfactants. The inorganic nanoclay has a general formula (Na,Ca)_0.33_(Al,Mg)_2_(Si_4_O_10_)(OH)_2_·*n*H_2_O. Examples of modified nanoclay are Cloisite^®^ 30B (Southern Clay Products) modified with 30 wt % methyl dihydroxyethyl tallow ammonium and Nanomer^®^ I.44P (Nanocor) modified with 35–45 wt % dimethyl dialkyl (C14–C18).

**Figure 7:**
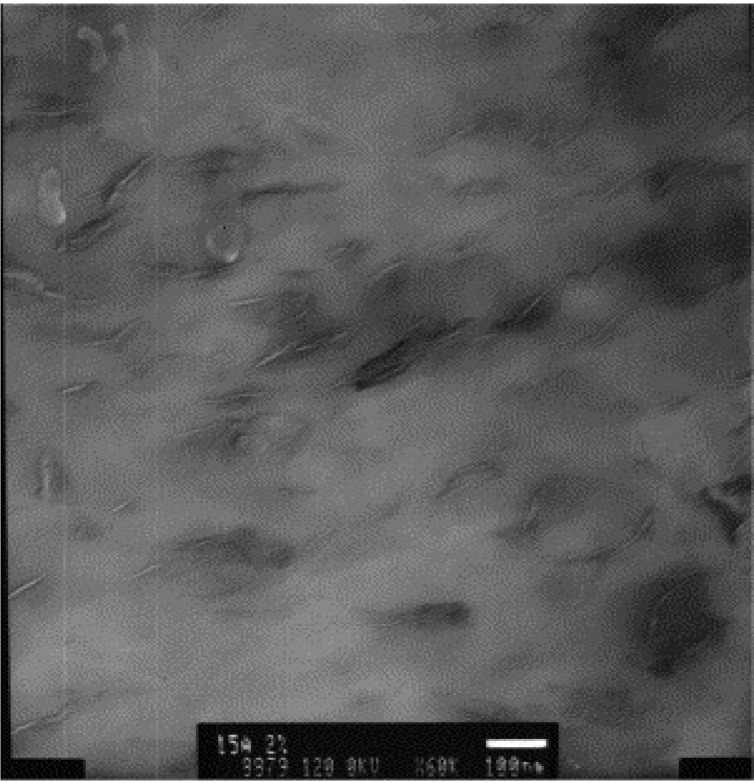
TEM image of precipitated 2 wt% Cloisite^®^ 15A/PVDF after hot-pressing into a film. The exfoliated silicate layers appear as sharp lines on a grey background from the PVDF matrix (Reprinted with permission from [81]. Copyright 2006 Elsevier).

During membrane fabrication, dispersing the nanoclay in solvent with prolonged stirring or ultrasonication is often adopted [46,81]. Solvent is used to swell up the individual layers such that polymer intercalation can occur in the galleries of the dispersed clay [77]. Figure 7 shows the exfoliated nanoclay platelets randomly distributed in the PVDF matrix.

##### 2.2.3.1. Mechanical Enhancement

The addition of nanoclay changed the mechanism of PVDF nucleation and promoted the shift to β-phase in the PVDF matrix [82]. Shah *et al*. [55] proposed that this change was linked to the mechanical enhancement in PVDF/nanoclay composites. Commercially available unmodified sodium montmorillonite (NCMU) and bis(hydroxyethyl) methyltallowammonium ion-exchanged montmorillonite (NCM) nanoclays were dispersed within PVDF to 5 wt % in a high energy mixer and melt extruded at 200 °C. For the PVDF/NCM material, tensile tests showed that the Young’s modulus increased from 1.3 to 1.8 GPa and the elongation at break increased from 20% to 140%. This inferred significant increase in toughness of ~700% higher than pure PVDF material. The authors suggested that nucleation of the fibre-like PVDF β-phase on the faces of individual silicate layers of the nanoclay brings about a structure which is more favourable to plastic flow under applied stress. This results in a more efficient energy-dissipation mechanism in the nanocomposites to delay cracking.

As membranes, PVDF/nanoclay nanocomposite materials also demonstrated improved mechanical properties [83,84,85,86]. Among four different types of nanoclays Cloisite^®^ Na+ (unmodified clay), Cloisite^®^ 15A (modified with 43 wt % dimethyl, dehydrogenated tallow, quaternary ammonium), Cloisite^®^ 20A (modified with 38% dimethyl, dehydrogenated tallow, quaternary ammonium), and Cloisite^®^ 30B (modified with 30 wt % methyl dihydroxyethyl tallow ammonium), maximum tensile strength was observed for the flat sheet membrane with 1 wt % of Cloisite^®^ 15A prepared with an air exposure time of 30 s before immersion into a water bath. The likely reason was because Cloisite^®^ 15A had the highest hydrophobicity which gave good affinity to the PVDF matrix [83].

Lai *et al*. [86] also noted by using different types of nanoclay, different aspects of the mechanical properties could be altered. Tensile strength increased from 3.8 MPa to 4.3 MPa with 5.08 wt % Cloisite^®^ 30B loading while break extension increased from 175% to 229% with 5.08 wt % Nanomer^®^ I.44P [modified with 35–45 wt % dimethyl dialkyl (C14–C18) amine] nanoclay loading. Other than the energy-dissipation mechanism induced by the PVDF β-phase, another reason for the improved mechanical properties can be attributed to the suppression of the macrovoids in the membranes with higher nanoclay loading.

Wang *et al*. [84] studied the effect of adding Cloisite^®^ 20A into PVDF hollow fibre for direct contact membrane distillation (DCMD) applications. Cloisite^®^ 20A was mixed with PVDF in NMP and ethylene glycol (EG) and the membranes were fabricated using the dry-jet wet phase inversion mechanism by using water as both internal and external coagulants. The ratio of dope was PVDF/NMP/Cloisite^®^ 20A/EG (10.0/74.7/3.3/12.0). The PVDF/20A membranes had lower ductility (extension at break) and stretch resistance (tensile stress), but increased stiffness (Young’s modulus) compared to the unmodified membranes. Unlike neat PVDF fibres which collapsed under long operation time, nanocomposite membrane was able to withstand the desalination test over 220 h with stable vapour flux. This study also showed the inclusion of clay particles enhanced long-term mechanical stability.

##### 2.2.3.2. Abrasion Resistance

Peng *et al*. [62] studied the tribological properties, including the abrasive wear resistance, of PVDF/nanoclay nanocomposite. 1–5 wt % of Nanomer^®^ I34TCN (modified with 25–30 wt % methyl dihydroxyethyl hydrogenated tallow ammonium) was melt extruded with PVDF at 190 °C, 180 rpm. It was observed that low nanoclay loading (1–2 wt %) had the highest ductility and impact strength as nanoclay can act as a temporary crosslinker to the polymer chains given their size and mobility are comparable. This provides localized regions of increased strength and inhibits the development of cracks and cavities. Nanocomposite at low nanoclay loading also had the lowest friction coefficient and wear rate. The author postulated that the shifting of the PVDF crystal phase induced by nanoclay addition increased the binding energy between macromolecules and improved abrasion resistance, as the material was less likely to peel off.

It was observed that nanoclay changed PVDF crystal phase and the PVDF/nanoclay nanocomposites demonstrated improvements to properties such as increases in toughness, strength and abrasion resistance. The incorporation into PVDF had benefits not only in providing physical reinforcement to the polymer network, but also acting as morphology directors by stabilising a metastable or conventionally inaccessible polymer phase, or introducing new energy dissipation mechanisms [55,87]. This led to enhanced toughness of the nanocomposites and greater abrasion resistance. It is evident that nanoclay has potential in improving these physical properties and hence the durability of PVDF membranes.

**Figure 8:**
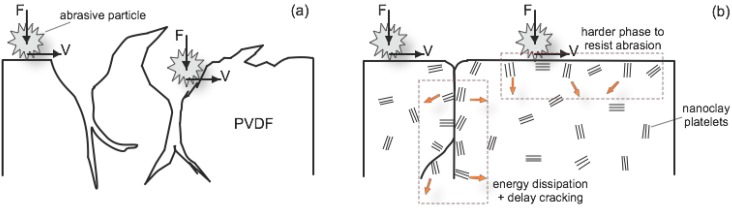
Proposed model for abrasion of (**a**) unmodified membrane and (**b**) the mechanically stabilized PVDF/nanoclay membrane (Reprinted with permission from [86]. Copyright 2014 Elsevier).

Using an in-house developed setup involving shaking hollow fibres in an abrasive slurry and periodically measuring bubble point for skin layer breakthrough, Lai *et al*. [86] evaluated the abrasion resistance of PVDF membranes reinforced with nanoclay. The result showed that the membrane with an initial 5.08% loading of Nanomer^®^ I.44P had improved abrasion resistance, lasting three times longer than the control membrane with no nanoclay addition. A model was proposed for the enhanced abrasion resistance of the nanocomposite membrane materials as shown in Figure 8. This incorporated both the energy dissipation mechanism due to PVDF phase change, as well as the presence of nanoclay which acted as a “harder” phase to the PVDF matrix which was now less exposed to abrasive wearing as the nanoclay resisted the wear with its enhanced mechanical strength [88].

##### 2.2.3.3. Flux Performance

PVDF/nanoclay membrane showed slightly lower water vapour flux than neat PVDF membrane during DCMD operation [84]. At inlet temperatures of 81.5 °C (3.5 wt % NaCl aqueous solution) and 17.5 °C (fresh water distillate), flux of the composite and the neat membrane was 79 kg/m^2^·h and 84 kg/m^2^·h, respectively. The drop in flux could be due to increases in tortuosity and thermal conductivity that acts to lower the thermal efficiency of the DCMD process. Despite this drawback, the composite membrane demonstrated enhanced mechanical performance especially for long operation times as stated in Section 2.2.3.1.

In MF/UF application, PVDF/nanoclay membranes also demonstrated lower pure water permeability compared to the control membrane [86]. One possible reason the control membrane having higher flux could be associated with its longer length of finger-like voids. While other types of nanoparticles shown in Section 2.2.1.2 improved water permeability, it could possibly those particles had higher hydrophilicity than nanoclay and thus led to better wetting and flow. Also, the nanoparticles used in those studies were spherical and much smaller than the membrane pore size. For instance, the size of TiO_2_ particles in the PVDF/TiO_2_ 1 wt % membrane was 25 nm and the average pore size was 0.18 μm [26]. For the PVDF/nanoclay membranes, the average pore size of the membranes was about 0.65 μm where the length of the nanoclay platelets was up to few hundreds nm. The reduced flux could be because some of the nanoclay particles were blocking the pores and/or increasing the tortuosity.

##### 2.2.3.4. Fouling Resistance

A laboratory *N*-vinlpyrrolidone modified MMT improved the anti-fouling properties of PVDF membrane when tested with BSA fouling solution due to changes of surface hydrophilicity and morphologies [27]. The anti-fouling properties of PVDF nanocomposites using commercial nanoclay have yet to be investigated.

A summary of the major findings of selected PVDF nanocomposite flat sheet and hollow fibre membranes is listed in Table 3. Currently there is more work done on flat sheet membranes than hollow fibres likely due to the simpler setup procedures. Although flat sheet membranes can be used to evaluate water treatment performance including water permeability and fouling profiles, hollow fibre is the more common membrane format used in water treatment and as such, studies performed on hollow fibres would be more relevant and applicable for water filtration uses. It was noted that the majority of the water treatment membrane studies focused on antifouling fouling performance using nanoparticles including TiO_2_ and SBA-15. While fouling is one of the durability issues mentioned in Section 1.2 and nanocomposite membranes have shown considerable improvement, fewer works focused on mechanical strength and abrasion resistance. Both PVDF flat sheet and hollow fibre membrane incorporated with commercial nanoclay have shown improved mechanical strength [83,84,85,86]. While SBA-15 [22], ZnO [28] and combination of TiO_2_/Al_2_O_3_ [67] also showed better mechanical performance than neat PVDF membrane, nanoclay had the advantages of low cost, commercial availability and effectiveness with low loading. Although SBA-15 was promising for both anti-fouling and mechanical improvement, it was required to be synthesized in-house and thus complicated the membrane preparation process. As for ZnO and TiO_2_/Al_2_O_3_, a much higher loading was often required compared to nanoclay. ZnO used by Liang *et al*. [28] was 6.7%–26.7% by weight of PVDF while Han *et al*. [67] used 3% by weight of dope which was equivalent to 17% by weight of PVDF. These were both considerably more than the 1 wt % Cloisite^®^ nanoclay (by weight of PVDF) used by Wang *et al*. [84]. Cost wise, nanoclay is more attractive and effective. The smaller loading of nanoclay is likely due to its ability to exfoliate and become finely dispersed in the polymer matrix. PVDF hollow fibre membranes incorporated with nanoclay also demonstrated enhanced abrasion resistance [86]. While nanocomposite membranes with nanoclay have the potential to be a practical and economical solution for improved physical durability, more work is needed for optimization the flux and to evaluate its fouling resistance particularly in the presence of additives such as PEG and PVP. Although there is no work on nanocomposite PVDF membranes dedicated to fibre breakage issue, the mechanical enhancement by various nanofillers is evident for their ability to resolve this issue. Further work is required to translate the reported mechanical improvements to other issues related to durability in future research.

Despite the identified progress in the use of nanofillers for improved membrane properties, there is little mentioned about the nanofillers becoming dislodged from the polymer matrix during filtration operation. Lai *et al*. [86] observed there was loss of nanoclay during the membrane fabrication process and this may infer nanoparticles can leach out from the membrane during filtration and become a source of contamination themselves. A study observing if gradual loss of nanofillers occurs, highlighting the loss rate into the treated water and the possible health/environmental risks, is therefore needed.

**Table 3:** Summary of selected PVDF nanocomposite membranes.

Nanofiller added	Type	Application	Casting condition	Observed changes	Ref.
40% TiO_2_ by weight of PVDF	Flat sheet	Mechanical support for composite membrane	PVDF dissolved in DMAc with LiCl then mixed with TiO_2_ Quench bath medium: water	Stronger resistance to compaction under pressure of 30 bar. Decrease of pore volume % improved from 83% to 17%. Produced better permeate quality with higher flux at elevated temperature and pressure (135 °C/6.5 bar) in the vapour permeation test.	[66]
TiO_2_, SiO_2_ and Al_2_O_3_ Ratio of dope: PVDF/DMAc/NMP/nanoparticles/PVP (18/59.2/14.8/3/5)	Hollow fibre	UF	24 h of mechanical stirring of PVDF and nanoparticles in DMAc/NMP/PVP at 25 °C then 1 h of ultrasonic stirring. Internal coagulant: 40 wt % ethanol aqueous solution at 60 °C External coagulant: water at 60°C	Increased dope viscosity Denser skin layer on the outer membrane surface Higher water permeability (increased from 82 L/m^2^·h·bar to 352 L/m^2^·h·bar with 2 wt % TiO_2_ & 1 wt % Al_2_O_3_) but varying BSA rejection percentages Tensile strength was improved from 1.71 MPa to 3.74 MPa with 2 wt % TiO_2_ & 1 wt % Al_2_O_3_	[67]
0.12–0.72 wt % SBA-15 by weight of PVDF	Flat sheet	UF	PVDF dissolved in DMAc and mixed with PVP and SBA-15 at 60 °C Quench bath medium: water	Improved mechanical properties: tensile strength increases from 0.151 MPa to 0.183 MPa (0.54 wt %); strain-at-break increases from 22.6% to 49.4% (0.36 wt %) Pure water flux increased from 372 L/m^2^·h·bar to 502 L/m^2^·h·bar (0.36 wt %) Ratio of permeate flux decline (flux ratio from start of filtration over set time) reduced from 24.4% to 15.5% (0.72 wt %) indicating antifouling property	[22]
1 wt % of Cloisite^®^ Na+ or 1 wt % of Cloisite^®^ 15A or 1 wt % of Cloisite^®^ 20A, or 1 wt % of Cloisite^®^ 30B by weight of PVDF	Flat sheet	Lithium-ion battery	PVDF dissolved in DMF at 70 °C then mixed with clay/DMF suspensions. Air retention time: 30 s or 60 s Quench bath medium: water	Longer retention time resulted in increase of finger-like macrovoids. PVDF/15A with 30 s in air had highest tensile strength (improved from 15 to 54 MPa)	[83]
Cloisite^®^ 20A Ratio of dope: PVDF/NMP/Cloisite^®^ 20A/EG (10.0/74.7/3.3/12.0)	Hollow fibre	DCMD	PVDF stirred with clay in NMP and EG mixture. Internal and external coagulants: water	Lower ductility and tensile stress but higher modulus. Enhanced long-term mechanical stability Reduced water vapour flux from 84 kg/m^2^·h to 79 kg/m^2^·h at inlet temperatures of 81.5 °C/17.5 °C (3.5 wt % NaCl/water)	[84]
0.88–5.08 wt % of Cloisite^®^ 30^B^ or 0.88–5.08 wt % of Nanomer^®^ I.44P by weight of PVDF	Hollow fibre	MF/UF	PVDF mixed with pre-dispersed nanoclay (dispersed with ultrasonication and a high shear hydrodynamic dispersion process) in NMP at 90 °C for 48 h and extruded with dry-wet spinning at 60 °C	Nanoclay shifted the PVDF crystalline phase from α-phase to β-phase Improved mechanical properties: tensile strength increased from 3.8 MPa to 4.3 MPa (5.08 wt % 30B); break extension increased from 175% to 229% (5.08 wt % I.44P). Improved abrasion resistance (5.08 wt % I.44P lasted three times longer). Reduced pure water permeability from 310 L/m^2^·h·bar to 182 L/m^2^·h·bar (2.61 wt % I.44P)	[86]

## 3. Conclusions

In this review, the need for physical durability and the state of art in the literature on PVDF nanocomposite membranes for durability and performance was reviewed. Various types of nanofillers including nanoparticles, CNT and nanoclays were evaluated for their effect on the membrane properties. While limited work has investigated the effect of CNT on PVDF membrane, other types of nanofillers have demonstrated improvement in flux, fouling resistance, mechanical strength and abrasion resistance with the correct choice of fillers and loadings. However, few studies have considered nanoparticles in the presence of additives such as PEG or PVP which may act to negate the influence of nanoparticles on surface properties. Overall, PVDF reinforced with commercially available nanoclay was fabricated as hollow fibre membranes for low pressure filtration applications showed a shift of the PVDF crystalline phase from α- to β-phase, and brought about a more efficient energy-dissipation mechanism in the nanocomposite membrane. With enhancement in both mechanical strength and abrasion resistance, nanoclay is the most promising nanoparticle for improving the physical durability of membrane.

## Abbreviations

BSA: bovine serum albumin
CNF: carbon nanofibers
CNT: carbon nanotubes
DCMD: direct contact membrane distillation
EG: ethylene glycol
MF: microfiltration
MFI: modified fouling index
MMT: montmorillonite
MOF: metal-organic frameworks
MWCNT: multi-walled nanotubes
NF: nanofiltration
NMP: 1-methyl-2-pyrrolidinone
NOM: natural organic matters
PEG: poly(ethylene glycol)
PP: polypropylene
PSf: polysulfone
PTFE: polytetrafluoroethylene
PVDF: poly(vinylidene fluoride)
PVP: poly(vinyl pyrrolidone)
RO: reverse osmosis
SBA-15: Santa Barbara Amorphous No. 15
SWCNT: single-walled nanotubes
UF: ultrafiltration
XRD: X-ray powder diffraction
